# Copying a soft lithography master mold using an inexpensive, hobby-use UV-curable resin

**DOI:** 10.1007/s44211-026-00920-2

**Published:** 2026-04-30

**Authors:** Kazuo Hosokawa, Hiroshi Kasuga, Hitoshi Ohmori

**Affiliations:** Materials Fabrication Laboratory, RIKEN Pioneering Research Institute, 2-1 Hirosawa, Wako, Saitama, 351-0198 Japan

**Keywords:** Poly(dimethylsiloxane), PDMS, Microfluidic chip, UV resin, Soft lithography, Fabrication

## Abstract

Poly(dimethylsiloxane) (PDMS) is the most widely used material for microfluidic chips because of its advantageous properties including easy fabrication through soft lithography, which means pattern transfer technique from microfabricated master molds. However, most of the master molds are made of a silicon wafer and a photoresist layer, and have two major disadvantages: (1) the fabrication process is complicated and requires specialized facilities, and (2) the fabricated master molds are fragile. Here, we describe a simple fabrication technique to produce a robust master mold by pattern transfer from an existing PDMS chip to an inexpensive, hobby-use UV-curable resin (UV resin). The process requires no specialized facilities or hazardous chemicals. The test pattern consisted of microchannels with a cross-section of 100 µm × 25 µm and a total area of 30 mm × 30 mm. This pattern was faithfully transferred from the PDMS surface to the UV resin. The inaccuracy of the solidified UV resin was evaluated to be smaller than the measurement error (< 0.2%). The UV resin mold was subsequently tested for the soft lithography of a next-generation PDMS chip. For successful soft lithography, the UV resin mold required heat treatment at 60 °C for at least 48 h prior to use. Without this treatment, substantial damage was observed on both the PDMS and the UV resin surfaces. This problem was probably caused by the inhibition of PDMS curing due to unreacted components of the UV resin remaining on the surface. The technique presented here provides a simple and low-cost route for the repetitive fabrication of identical PDMS microfluidic chips for various research fields.

**Graphical abstract:**

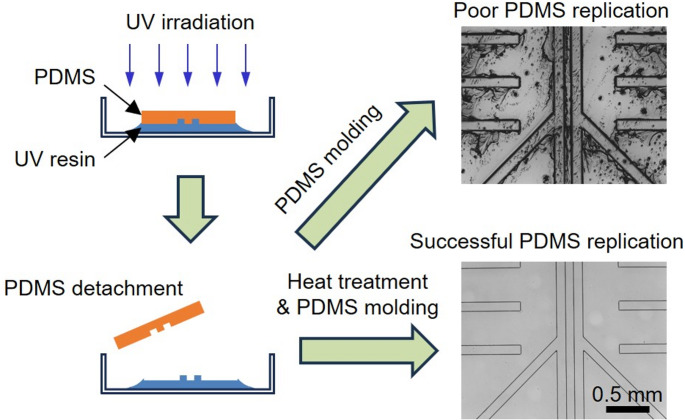

**Supplementary Information:**

The online version contains supplementary material available at 10.1007/s44211-026-00920-2.

## Introduction

Poly(dimethylsiloxane) (PDMS) is the most widely used material for the fabrication of microfluidic chips because of its chemical stability, optical transparency, mechanical flexibility, and easy fabrication. PDMS microfluidic chips are usually fabricated through soft lithography process, which means pattern transfer from a microfabricated master mold to a PDMS prepolymer [1–4]. In most cases, the master mold is fabricated from a silicon wafer and a layer of ultrathick photoresist (e.g., SU-8). Although these master molds are basically reusable after demolding the cured PDMS, they are often broken due to the brittleness of the silicon wafer and the tendency of delamination of the ultrathick photoresist layer. In such cases, refabrication of the master mold is time-consuming and requires cleanroom facilities. More robust master molds have been fabricated through alternative methods such as mechanical micromilling [5, 6] and three-dimensional (3D) printing [7–9]. These methods employ metal or plastic substrates, which are more rigid than silicon wafers. The resulting master molds are monolithic and therefore do not contain interfaces susceptible to delamination. However, these alternative methods generally have limitations in terms of spatial resolution and surface roughness, unless some advanced technologies—such as high-precision micromilling [10] or two-photon polymerization [7, 11]—are employed. These advanced technologies require sophisticated facilities, long fabrication time, or both.

For the facile fabrication of rigid and monolithic molds, another group of methods has been reported: replication of the microstructures from an existing PDMS surface onto a plastic material. As plastic materials, two-part polyurethane [7, 12], two-part epoxy [13, 14], and melted polycarbonate [15, 16] have been utilized. In addition to the robustness of the fabricated mold, an advantage of these methods is that multiple copies of the original mold can be produced from a single PDMS chip. In general, this copying process is simpler and less dependent on facilities than the aforementioned processes, namely, photolithography, mechanical micromilling, and 3D printing. In earlier studies, UV-curable resins (UV resins) were also used for the fabrication of soft lithography molds through pattern transfer from PDMS surfaces [17, 18]. However, the objective of these early studies was the fabrication of unconventional structures, such as sinusoidal waves [17] and small apertures [18], rather than the replication of an original master mold. More recently, 3D-printable UV resins have been tested for copying an original master mold, with the aim of identifying optimal post-treatment conditions for UV resin surfaces to enable successful PDMS soft lithography on those resin surfaces [8].

Herein, we describe a fabrication technique for producing a copy of a soft lithography mold—originally made from a silicon wafer and an SU-8 layer—using a UV resin. The UV resin was selected from inexpensive, hobby-use products intended for general consumers. We adopted a test pattern that was previously used in our study of microRNA analysis [19]. The test pattern had typical dimensions as a microfluidic chip: microchannels with a cross-section of 100 µm × 25 µm and a total area of 30 mm × 30 mm. A PDMS chip was fabricated by conventional soft lithography on the original mold, and the surface pattern of the PDMS chip was subsequently transferred to the UV resin to produce a copy of the original mold. The resulting copy was then used for fabrication of a next-generation PDMS chip. Under various process conditions, the fabrication results were evaluated based on their appearance. After optimization of the process conditions, the accuracy of the copy was quantitatively evaluated.

## Experimental

### Conventional soft lithography

**Fig. 1:**
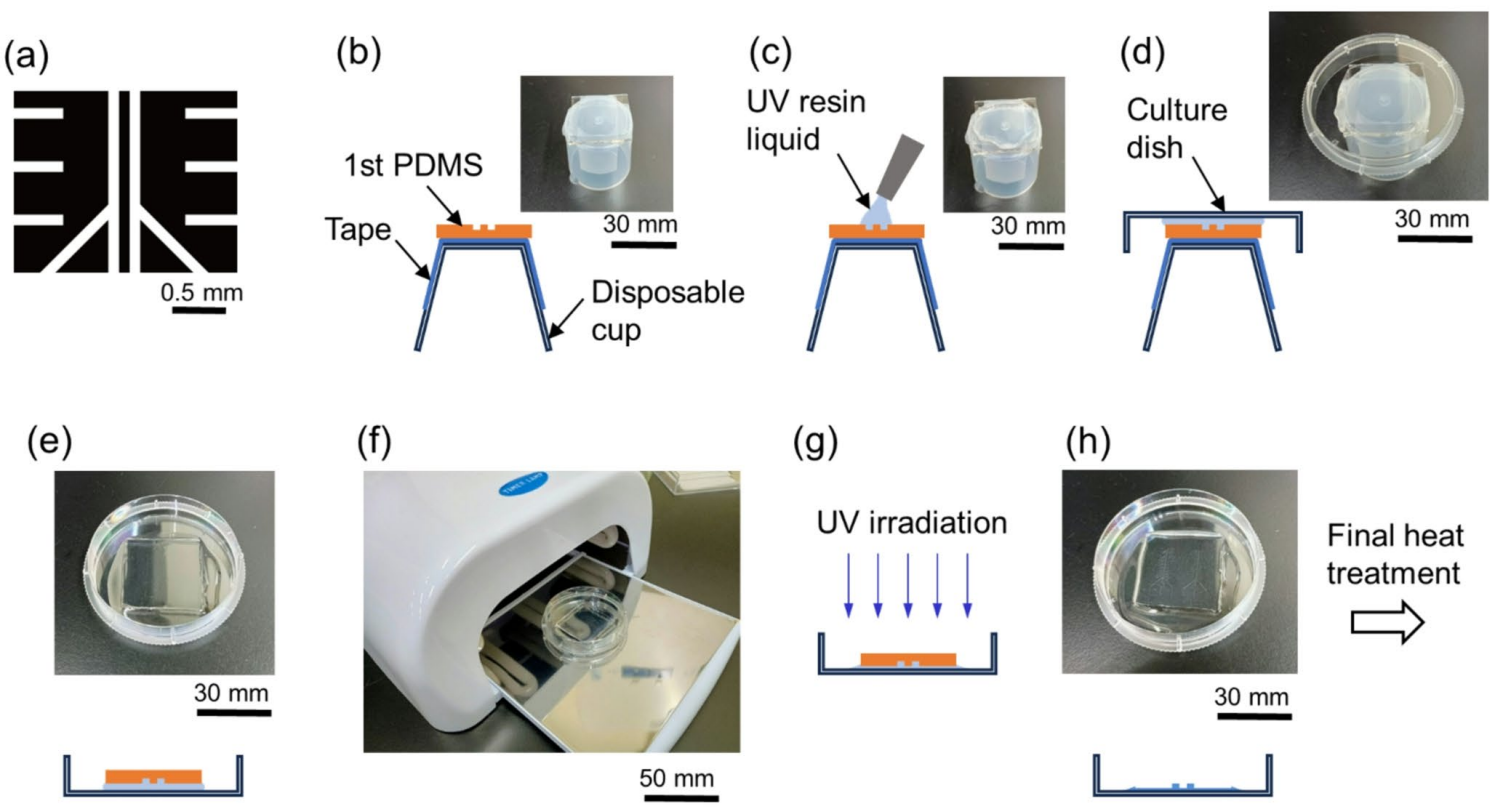
Summary of the fabrication protocol. **a** Central part of the test pattern. The entire pattern is shown in Fig. S1 in the SI. **b** The 1st PDMS was placed on a handmade stage. **c** The UV resin liquid was poured onto the surface of the 1st PDMS. **d** The UV resin liquid was spread using a culture dish. **e** The assembly from the stage to the culture dish was turned upside down, and the stage was removed. **f** The culture dish containing the UV resin and the 1st PDMS was placed in the chamber of a 36-W UV lamp. **g** UV irradiation was applied through the 1st PDMS for 20 min. **h** After detachment of the 1st PDMS from the solidified UV resin, the UV resin was heated in an oven at 60 °C for 48 h

Figure 1a shows the central part of the test pattern used in this work. The entire pattern is shown in Fig. S1 in the Supplementary Information (SI). This pattern was originally designed in our previous work [19]. All microchannels were 100 µm wide and 25 µm deep. The outer dimensions of the PDMS chip were 30 mm × 30 mm × 2 mm. As described previously [19], a conventional mold was fabricated using a silicon wafer and an ultrathick photoresist (SU-8 25, MicroChem). Onto the mold, a PDMS prepolymer (Silpot 184, Dow Chemical, base: curing agent = 10:1) was poured. Air bubbles in the PDMS prepolymer were removed in a vacuum chamber, and the prepolymer-filled mold was then incubated in an oven at 65 °C for 1 h. The solidified PDMS chip (“1st PDMS” hereafter) was peeled off from the mold. The 1st PDMS was reversibly bonded to a flat glass plate and further heated at 100 °C for 1 h to ensure complete curing.

### Fabrication of the UV resin mold

Figure 1b–h illustrate the steps of pattern transfer from the 1st PDMS to the UV resin. In advance, the 1st PDMS was degassed in the vacuum chamber at 10 kPa for at least 40 min so that the recesses on the 1st PDMS surface would be completely filled with UV resin liquid by the degas-driven, power-free pumping mechanism [20]. To elevate the position of the 1st PDMS, a stage was prepared using a disposable cup, which was covered with pieces of Scotch tape to prevent stiction of the 1st PDMS. The 1st PDMS was then placed on the stage with the patterned side facing upward (Fig. 1b). Onto the 1st PDMS, a drop (0.5–1 g) of UV resin liquid (Moon Drop, Padico) was poured (Fig. 1c), and immediately covered with the bottom of a ϕ60 mm culture dish (Nunc EasYDish, Thermo Fisher Scientific) (Fig. 1d). After spreading the UV resin liquid across the surface of the 1st PDMS, the entire assembly (from the stage to the culture dish) was quickly turned upside down, and the stage was removed (Fig. 1e). The backside of the 1st PDMS was gently pressed using the grip side of a tweezer to push out visible bubbles beyond the PDMS edges. To release possible stress in the 1st PDMS, the assembly (the culture dish, the UV resin liquid, and the 1st PDMS) was kept in a dark, level place at room temperature (~ 25 °C) for 20 min and then placed in the chamber of a UV lamp (Super Resin UV Crystal Lamp 36 W, Kiyohara, wavelength 365 nm) (Fig. 1f). UV irradiation was applied to the UV resin liquid through the 1st PDMS for 20 min (or 10 min in some experiments) (Fig. 1g). After cooling at room temperature for 10 min, the 1st PDMS was detached from the solidified UV resin, which formed a “UV resin mold” together with the culture dish (Fig. 1h). Finally, the UV resin mold was heated in the oven at 60 °C for 48 h (or 0–24 h in some experiments).

### Soft lithography using the UV resin mold

The UV resin mold was used for the fabrication of a next-generation PDMS chip (“2nd PDMS” hereafter) via soft lithography. Onto the UV resin mold, 5 g of PDMS prepolymer was poured. After the removal of air bubbles under vacuum, the PDMS prepolymer was incubated in the oven at 55 °C overnight (16–24 h). The cured 2nd PDMS was then peeled off from the UV resin mold.

### Evaluation of the fabrication results

The surfaces of the 1st PDMS, the UV resin mold, and the 2nd PDMS were observed using an optical microscope (Hisomet II, Union Optics). The microscope was equipped with a digital measurement system and was also used to measure the lateral dimensions of the 1st PDMS, the UV resin mold, and the 2nd PDMS. The vertical dimensions of the 1st PDMS and the UV resin mold were evaluated using surface profile plots obtained with a non-contact 3D measuring instrument (NH-3, Mitaka Kohki). The surface roughness of each sample was measured with a 3D surface profiler (VK-X3100, Keyence).

## Results and discussion

### Fabrication of the UV resin mold and the 2nd PDMS

We selected a UV resin and a UV lamp from consumer-use products, as specified in the Experimental section. These products are widely used for hobby purposes, such as the handcrafting of toys and accessories. They were relatively inexpensive: 1200 Japanese yen (¥1200) per 30 g for the UV resin and ¥4680 for the UV lamp (as of February 2026). The UV resin mold was fabricated without the use of cleanroom facilities, specialized instruments, or hazardous chemicals. Through preliminary experiments, we observed that the final heat treatment (FHT, 60 °C) time and the UV irradiation time were important process parameters. Therefore, the effects of these parameters were mainly investigated.

We also optimized the curing condition for the 2nd PDMS. Under the same curing condition used for the 1st PDMS (65 °C for 1 h), we sometimes observed minor residual PDMS on the mold surface due to stiction, because the UV resin mold surface had undergone no special treatment for mold release. We found that lowering the curing temperature to 55 °C was effective to solve this problem. Another possible solution would be treatment of the mold surface such as Cr/Au coating [14].

**Fig. 2:**
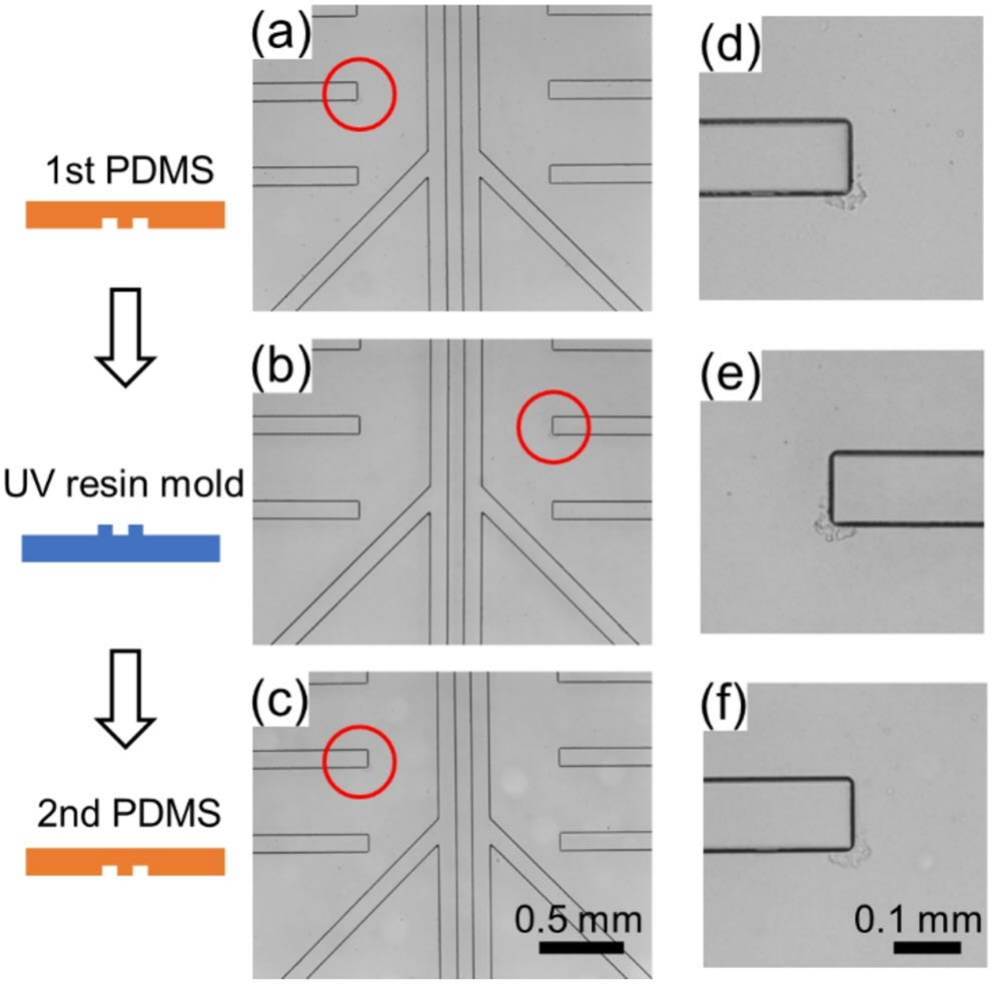
Bright-field images of the fabrication results. **a** The 1st PDMS. **b** The UV resin mold. **c** The 2nd PDMS. **d**–**f** Close-up views of the circled regions in (**a**–**c**), respectively. UV resin processing conditions: UV irradiation time, 20 min; final heat treatment (FHT) time, 48 h

Figure 2 shows bright-field images of the 1st PDMS, the UV resin mold, and the 2nd PDMS. The UV resin mold was replicated from the 1st PDMS under the optimal conditions: 20 min of UV irradiation and 48 h of FHT. The replication results appeared excellent. In fact, even minor irregularities in the 1st PDMS were faithfully transferred to the corresponding positions on the UV resin mold, as shown in Fig. 2d and e. The replication process caused no damage to the surface of the 1st PDMS. Indeed, throughout this study, we repeatedly used only two individual chips as the 1st PDMS; more than ten replication processes resulted in no observable damage to the PDMS surfaces. The replication results were consistently excellent immediately after the fabrication of the UV resin molds, regardless of the UV irradiation time or the FHT time. Differences arising from these parameters became apparent only after the fabrication of the 2nd PDMS, as described in the following subsections. Under the optimal conditions, the 2nd PDMS was fabricated without any issues (Fig. 2c and f). Incidentally, when the FHT time was further extended (e.g., ~ 72 h over a weekend), no harmful effects were observed. Here, “optimal” refers to the shortest FHT time (and UV irradiation time) that was confirmed to function without problems.

In general, there are two methods for demolding the cured PDMS from this type of dish-based master molds. In Method 1 (demold and cut), the whole, disk-shaped PDMS is demolded, and the unnecessary peripheral parts are cut off. In Method 2 (cut and demold), the cured PDMS is cut into the defined size (30 mm square in this case) on the mold, and only the cut part is peeled off. Both methods have their own advantages and disadvantages. We basically use Method 1, because the use of Method 2 may damage the mold surface with the cutter blade during the cutting process. As a result, we used a single UV resin mold at least 50 times in another research project without observing any deterioration in and around the patterned area, although the outermost area had many scratches made by tweezers at the beginning of the demolding process. The UV resin molds are likely to have sufficient mechanical robustness for ordinary research purposes.

Functionality of the 2nd PDMS as a microfluidic chip was confirmed through rudimentary experiments involving the observation of laminar flow using a fluorescent dye (Fig. S2 in the SI) and irreversible bonding to a glass surface (Fig. S3 in the SI). We also tested three other UV resin products available in Japan under the same conditions: 20 min of UV irradiation and 48 h of FHT. With all the tested products, both the UV resin molds and the 2nd PDMS chips were successfully fabricated without visible defects (Fig. S4 in the SI). These results imply that the optimal parameters identified in this study would be effective for a wide range of UV resin products.

### Effect of the FHT time

**Fig. 3:**
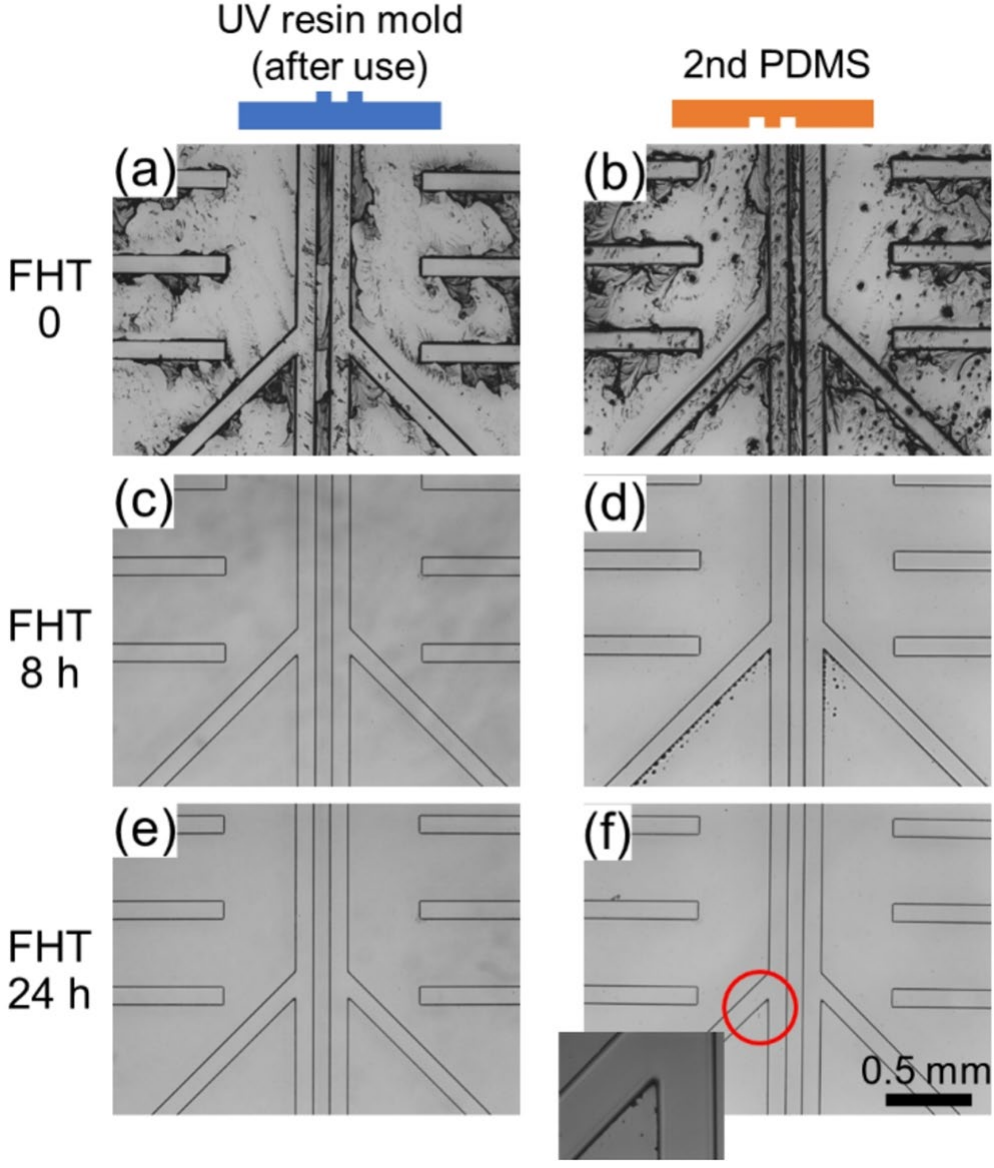
Bright-field images of the fabrication results obtained with 20 min of UV irradiation and various FHT durations. **a**, **b** No FHT. **c**, **d** 8 h of FHT. **e**, **f** 24 h of FHT. **a**, **c**, **e** The UV resin molds after use. **b**, **d**, **f** The 2nd PDMS. The inset in (**f**) shows a close-up view of the circled region

Figure 3 shows bright-field images of the UV resin molds after being used for soft lithography, and the corresponding 2nd PDMS chips obtained with various durations of the FHT. The UV irradiation time was fixed to 20 min. Without the FHT, both the UV resin mold and the 2nd PDMS surfaces exhibited unacceptable damage (Fig. 3a and b). A substantial amount of PDMS appeared to have remained on the mold surface. Similar results were reported for 3D-printed UV resins in a previous study [8], where the problem was attributed to PDMS curing inhibition caused by unreacted components such as monomers or photo-initiators. We hypothesize that the damage observed in Fig. 3a and b was caused by a similar mechanism, because this phenomenon was widely observed with various 3D-printed UV resins [8], which were probably solidified through photochemical reactions similar to that of the UV resin used in this work. The FHT is considered to be effective in removing these inhibitors from the UV resin surface by evaporation. With 8 h or 24 h of FHT, no damage was observed on the mold surfaces (Fig. 3c and e), while some defects were still present on the 2nd PDMS surfaces (Fig. 3d and f). In the case of 24 h FHT, the defects on the 2nd PDMS surface were very minor (Fig. 3f, inset) and may be acceptable for many applications.

### Effect of the UV irradiation time

**Fig. 4:**
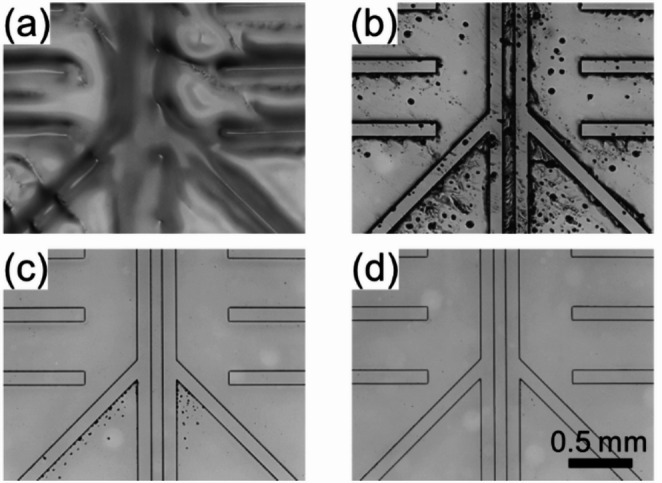
Bright-field images of the 2nd PDMS replicated from the UV resin molds which had been fabricated with 10 min of UV irradiation and various FHT durations. **a** No FHT. **b** 8 h of FHT. **c** 24 h of FHT. **d** 48 h of FHT

We also conducted similar experiments to those described above using a shorter duration of UV irradiation: 10 min. The results are shown in Fig. 4. In all cases, the damage observed on both the UV resin mold and the 2nd PDMS surfaces was more severe than that obtained with 20 min of UV irradiation and the corresponding FHT durations. In fact, without FHT (Fig. 4a), curing of the 2nd PDMS was inhibited to such an extent that even adjusting the microscope focus was difficult. With 8, 24, and 48 h of FHT (Fig. 3b, c, and d, respectively), the surface damage appeared similar to that observed with 20 min of UV irradiation and one-step shorter FHT durations: 0, 8, and 24 h, respectively. These results are consistent with the hypothesis that inhibition of PDMS curing was caused by unreacted components remaining after the photochemical reaction, because the reduction in UV dose would increase the amount of these unreacted components. Further extension of the FHT time (> 48 h) would likely yield successful results even with 10 min of UV irradiation. It is worth noting that the manufacturer of this UV resin product recommends UV irradiation time of 2–4 min for each side (i.e., 4–8 min in total). In this work, we adopted longer UV irradiation time, considering the fact that the UV rays were irradiated through the 1st PDMS.

### Accuracy of the UV resin mold

**Fig. 5:**
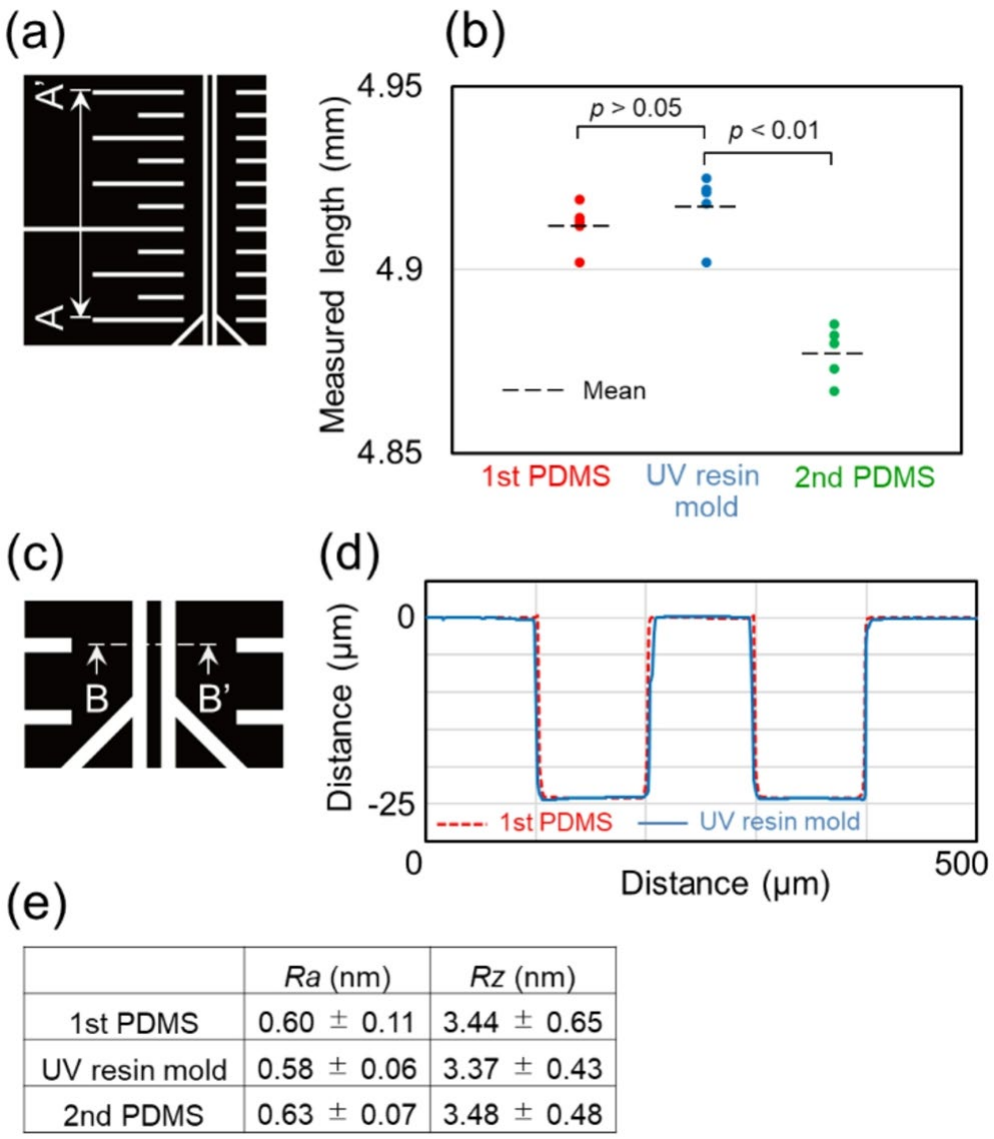
Evaluation of the accuracy of the UV resin mold. UV resin processing conditions: UV irradiation time, 20 min; FHT time, 48 h. **a** The length of section A-A’ was measured. This figure represents the PDMS side. For the UV resin mold, the corresponding part was measured. **b** Measurement results. **c** Surface profile plots of section B-B’ were obtained. **d** Superimposed surface profile plots of the 1st PDMS (red dashed line) and the UV resin mold (blue solid line). **e** Surface roughness parameters at the center of the samples, expressed as mean ± 1 standard deviation (*n* = 5). *Ra*, arithmetic mean height; *Rz*, maximum height; evaluation length, 0.4 mm

We quantitatively evaluated the accuracy of the UV resin mold fabricated under the optimal conditions: 20 min of UV irradiation and 48 h of FHT. As a representative lateral dimension, we measured the length specified in Fig. 5a (designed value: 5 mm). The measurements were repeated on single samples of the 1st PDMS, the UV resin mold, and the 2nd PDMS five times each. The results are summarized in Fig. 5b. Although we had anticipated a shrinkage of the UV resin, the mean measured length of the UV resin mold was slightly greater than the corresponding length of the 1st PDMS. However, the difference was very small (0.006 mm, corresponding to 0.12% of the total length) and was evaluated as non-significant by a *t*-test. The 2nd PDMS exhibited a significant shrinkage of 0.82%. This shrinkage is somewhat smaller than the typical 1–2% shrinkage resulting from our conventional soft lithography recipe (65 °C for 1 h followed by 100 °C for 1 h, data not shown). This difference was probably caused by the lower curing temperature of the new recipe (55 °C). Compared to these inaccuracies of PDMS chips, which do not cause problems in most applications, the inaccuracy observed for the UV resin mold in this study is even smaller and can be considered negligible.

To evaluate the accuracy in the vertical direction, surface profile plots of section B-B’ shown in Fig. 5c were obtained using the non-contact 3D measuring instrument. The results are presented in Fig. 5d. Between the two profile plots, no distinct discrepancy was observed. We also measured the surface roughness of each sample (Fig. 5e). The measured roughness parameters had no significant difference among these samples. Overall, no issues regarding the accuracy of the UV resin mold were identified for the tested surface pattern, which had characteristic lengths on the order of tens to hundreds of micrometers. These dimensions are relevant to many microfluidic applications.

## Conclusions

We have detailed a fabrication protocol for producing a copy of a soft lithography master mold using a hobby-use UV resin product, and presented the fabrication results under various conditions. The fabrication process required low cost and minimal facilities. The fabrication time was 1–2 h of hands-on time followed by 48 h of waiting time, which can be used for parallel processing of multiple molds. The inaccuracy of the UV resin mold was found to be smaller than the measurement error. The technique described here would be useful for producing robust and identical copies of original soft lithography master molds.

In general, consumer-use products—in comparison with industrial- or research-use products—have advantages such as low cost and ease of purchase in small quantities. Conversely, consumer-use products often have disadvantages of limited information regarding their chemical and physical properties, and a higher risk of abrupt change in these properties or even discontinuation of production. In this study, we tested the fabrication protocol with a microfluidic chip of typical dimensions: microchannels of a cross-section of 100 µm × 25 µm and a total area of 30 mm × 30 mm. In the future, it would be worthwhile to test this fabrication protocol with smaller feature sizes, higher aspect ratios, and larger total areas.

## Supplementary Information

Below is the link to the electronic supplementary material.

Supplementary Material 1

## Data Availability

The datasets generated during and/or analyzed during the current study are available from the corresponding author on reasonable request.
