# Supplementary material for: Copying a soft lithography master mold using an inexpensive, hobby-use UV-curable resin
Source: Anal Sci. 2026 Apr 30;42(8):787–93. doi: 10.1007/s44211-026-00920-2 (PMC13400689; doi:10.1007/s44211-026-00920-2)
Supplement: Supplementary file 1 — Supplementary Material 1 [file 44211_2026_920_MOESM1_ESM.docx]

**Supplementary Information**

**Copying a soft lithography master mold using an inexpensive, hobby-use UV-curable resin**

Kazuo Hosokawa^1^, Hiroshi Kasuga^1^, and Hitoshi Ohmori^1^

^1^Materials Fabrication Laboratory, RIKEN Pioneering Research Institute, 2-1 Hirosawa, Wako, Saitama 351-0198, Japan


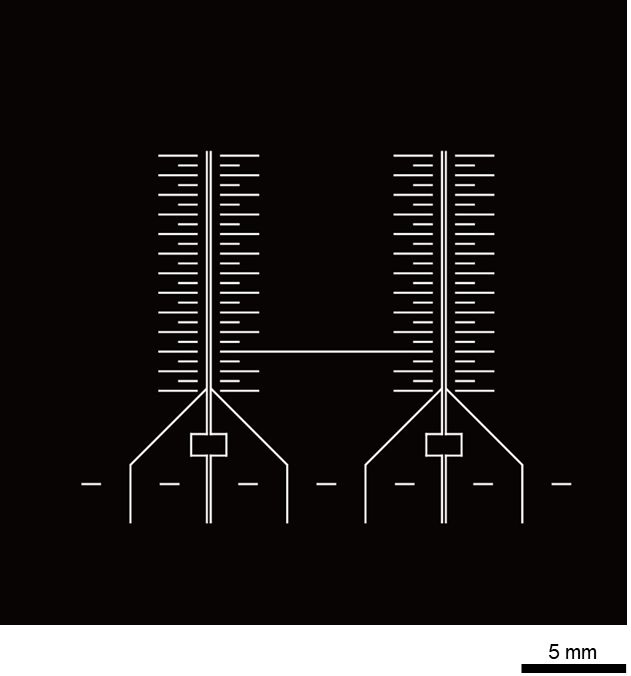


**Fig. S1** The test pattern used in this work. All the lines are 100 µm wide. There are two sets of double-Y microchannels. The horizontal lines worked as alignment marks in our previous work [1].

Reference

1. H. Arata, H. Komatsu, K. Hosokawa, M. Maeda, Plos One (2012). https://doi.org/10.1371/journal.pone.0048329


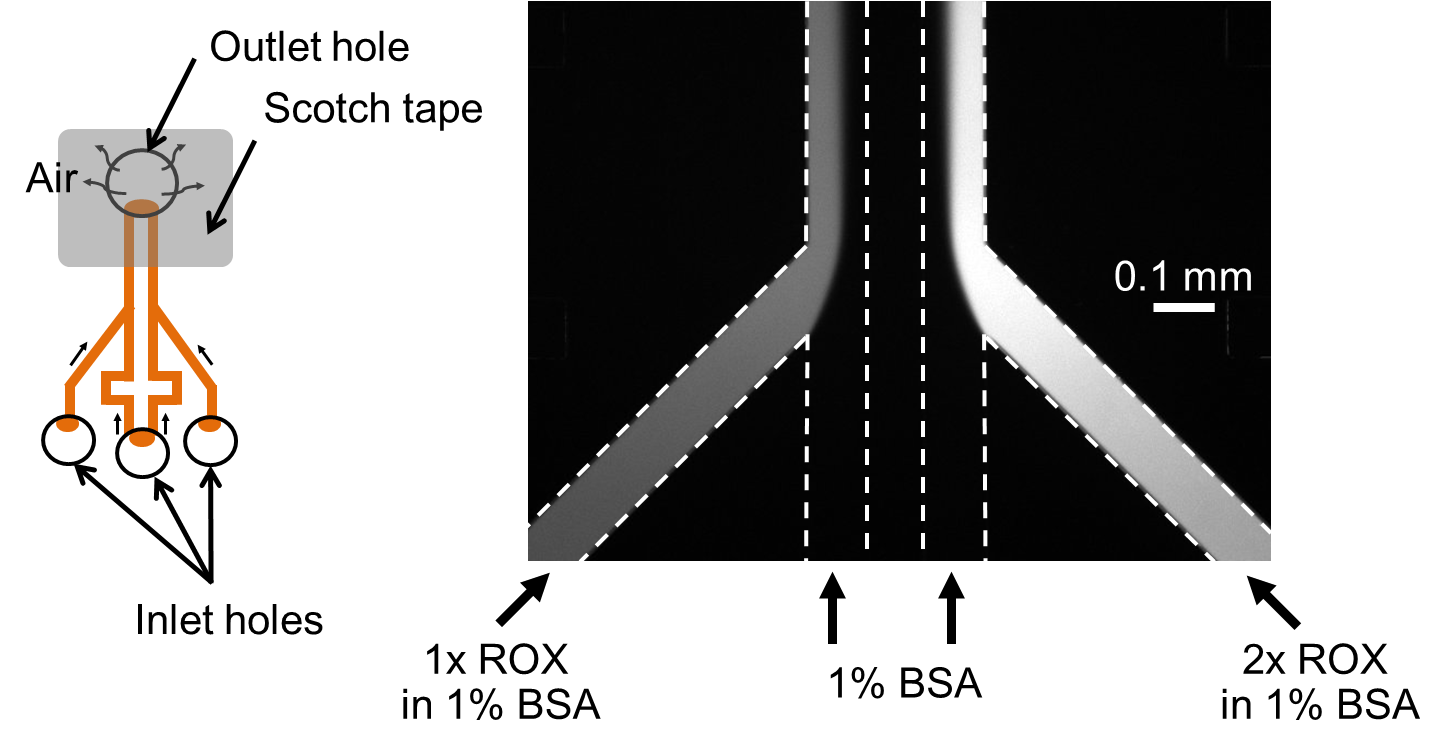


**Fig. S2** A demonstration experiment using the 2nd PDMS. Metal pipes were used to punch out inlet holes (ϕ2.5 mm) and an outlet hole (ϕ4 mm) in the 2nd PDMS. The PDMS was reversibly bonded to a flat glass plate to complete a microfluidic chip. Test solutions were prepared by mixing 50 mg/mL bovine serum albumin (BSA, Thermo Fisher Scientific), 50x ROX dye (Takara), and pure water. The BSA was supplemented to prevent the dye molecules from being adsorbed onto the PDMS walls. The microfluidic chip was degassed in a vacuum chamber at 10 kPa for at least 40 min. The outlet hole of the microfluidic chip was blocked using a piece of Scotch tape. The solutions specified in the figure were injected into the inlet holes. The solutions were pumped by the degas-driven power-free pumping mechanism [1]. The fluorescence image was acquired using a fluorescence microscope (Ti-U, Nikon) with a filter block (excitation 535 nm, emission 590 nm).

Reference

1. K. Hosokawa, Anal. Sci. (2021). https://doi.org/10.2116/analsci.20SCR04


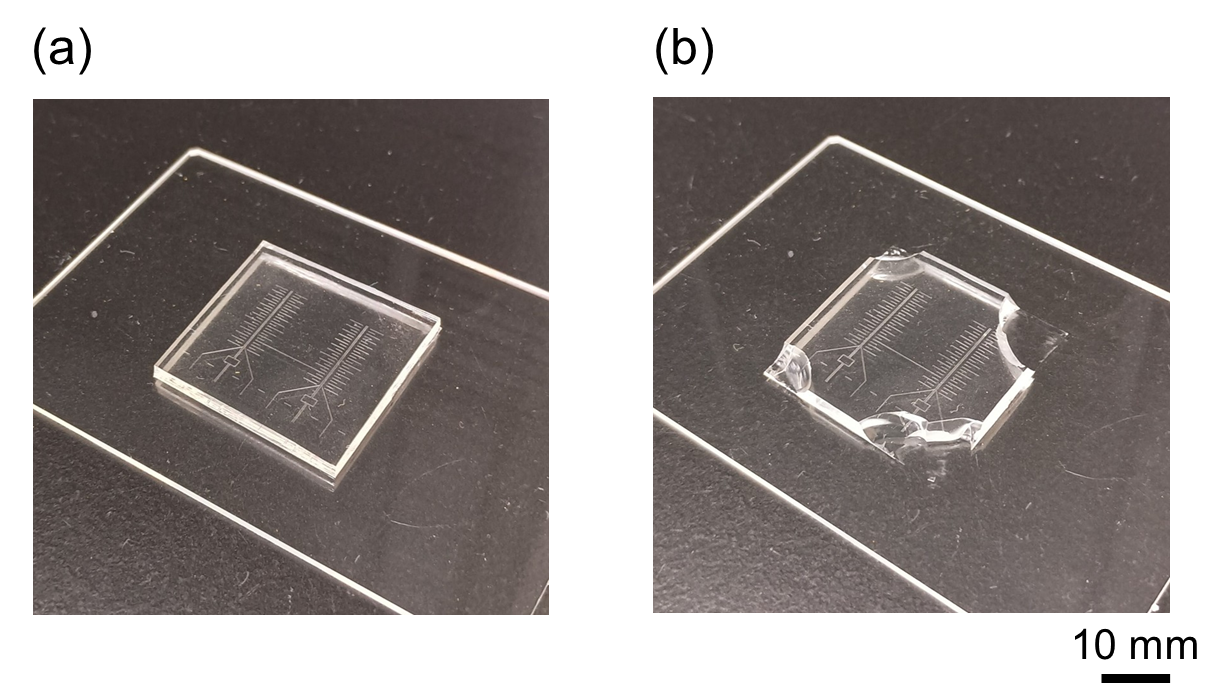


**Fig. S3** Irreversible bonding of the 2nd PDMS to a glass plate. Using an atmospheric-pressure plasma generator (P500-SM, Sakigake), air plasma was applied to the surfaces of the 2nd PDMS and a glass slide (S9112, Matsunami) for 30 s each. The two surfaces were brought into contact with each other, and the assembly was heated in an oven at 100 °C for 2 h. (a) Before the delamination test. (b) After the delamination test. The 2nd PDMS could not be peeled off without breaking it. This result indicates that the bonding strength was comparable to or higher than the bulk strength of the PDMS.


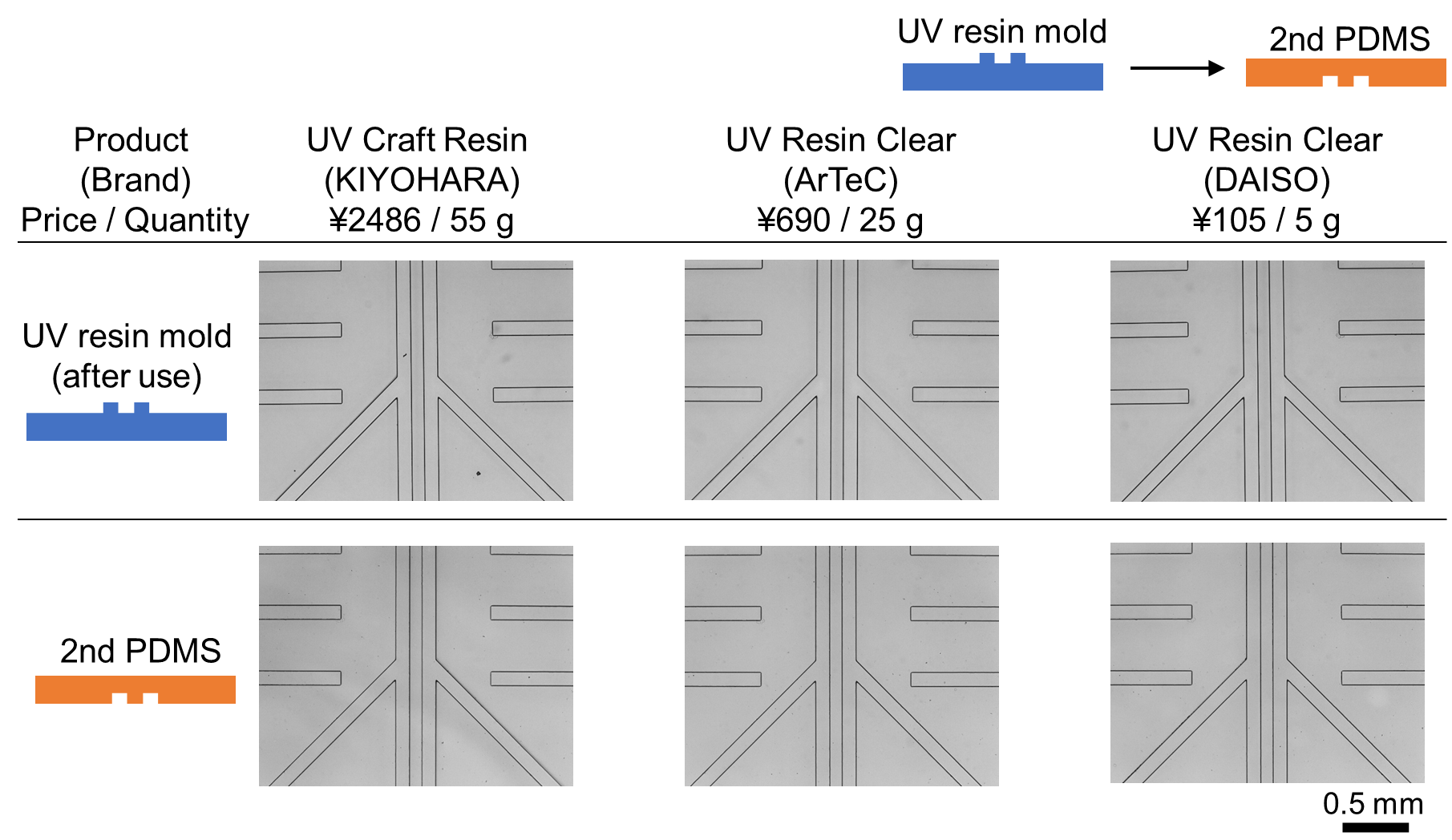


**Fig. S4** Bright-field images of the UV resin molds made from three different hobby-use UV resin products available in Japan (middle row) and the 2nd PDMS chips replicated from the corresponding UV resin molds (lower row). The product information (upper row) was as of February 2026. UV resin processing conditions: UV irradiation time, 20 min; final heat treatment time, 48 h.
